# Monitoring of Fiber-Reinforced Composite Single-Lap Joint with Electromechanical Impedance of Piezoelectric Transducer

**DOI:** 10.3390/ma12193241

**Published:** 2019-10-04

**Authors:** Jianjian Zhu, Xinlin Qing, Qijian Liu, Xiao Liu, Yishou Wang

**Affiliations:** School of Aerospace Engineering, Xiamen University, Xiamen 361002, China; zhuaero@stu.xmu.edu.cn (J.Z.); xinlinqing@xmu.edu.cn (X.Q.); saytdgdu6@stu.xmu.edu.cn (Q.L.); 34720180155075@stu.xmu.edu.cn (X.L.)

**Keywords:** fiber-reinforced composites, single-lap joint, quasi-static behavior, electromechanical impedance, piezoelectric transducer, structural health monitoring

## Abstract

The single-lap joint of fiber-reinforced composites is a common structure in the field of structure repair, which has excellent mechanical properties. To study and monitor its quasi-static response behavior under external load, two methodologies called effective structural mechanical impedance (ESMI) and reduced-ESMI (R-ESMI) are presented in this article. A two-dimensional electromechanical impedance (EMI) model for a surface-bonded square piezoelectric transducer (PZT) is adopted to extract more sensitive signatures from the measured raw signatures. There are two major advantages of the monitoring scheme based on ESMI and R-ESMI signatures: (1) excellent monitoring results with less signatures to process, (2) the ability to monitor the quasi-static behavior of a single-lap joint with previously ignored susceptance signatures. Combining the extracted ESMI signatures with the index of root-mean-square deviation, the quasi-static behavior of single-lap joints can be effectively quantified. To test the effectiveness of ESMI methodology, verifying experiments were conducted. The experimental results convincingly demonstrated that the presented ESMI and R-ESMI methodologies have good feasibility in monitoring the quasi-static behavior of a fiber-reinforced composite single-lap joint. The proposed method has potential application in the field of structural health monitoring (SHM).

## 1. Introduction

Adhesive joint structures are widely applied in the aerospace, automotive, and marine industries for their excellent mechanical properties and lightweight advantages. A single-lap joint is one of the most commonly used adhesive structures due to its easy and cost-effective production, especially in the field of fiber-reinforced composite repair. However, with the increase in service time, single-lap joints may suffer damages under the action of external adverse factors, such as impact and environmental impairment. The occurrence of these damages may severely reduce the mechanical performance of the joint structure [1,2,3,4]. To solve this problem, some advanced techniques are adopted to detect/monitor the damage inside the structures, such as non-destructive testing (NDT) and structural health monitoring (SHM).

Conventional NDT techniques contain some typical approaches, such as visual inspection, radiography, magnetic particle testing, eddy current testing, and ultrasonic testing [5]. Despite several NDT approaches successfully put into practice in some scenarios, most of them are usually performed offline and require expensive facilities. SHM techniques are developed from conventional NDT to some extent, and they are simpler, smarter, and characterized by online monitoring. This is the main reason for SHM being a rising and burgeoning technique distinguished from traditional NDT methods. As a significant method for determining the integrity of structures, SHM involves the use of multidisciplinary fields including sensors, materials, signal processing, system integration, and signal interpretation [6]. Active SHM approaches are widely used in many industries, including ultrasonic guided wave (GW) and electromechanical impedance (EMI). GW is a kind of stress wave that can propagate along waveguides such as pipes and plate-like structures. Although many researchers applied the GW technique to detect the damage of composite panels and stiffened structures on delamination, fatigue damage, repair patch disbond, and internal damage of concrete structures using damage indicators or damage imaging [7,8,9,10,11,12,13,14,15,16,17,18,19,20,21], only a few studies aimed at lap joints to investigate their failure characteristics [1,2,3,4,22,23,24]. Nevertheless, when used for detecting complex structures, the scattering properties of GW are usually very complicated and difficult to analyze; thus, another active monitoring technique called EMI is taken into consideration. The EMI method was developed by many researchers and used for evaluating the damages inside composite or metal structures. Ai et al. [25,26] adopted the EMI technique for the damage monitoring of civil structures with surface-bonded or embedded piezoelectric transducers (PZT). Annamdas et al. [27,28] proposed a three-dimensional EMI interaction model, but this theoretical model is too complex to be applied in engineering scenarios. Bhalla et al. [29,30] developed a simplified EMI model, which has potential application in the SHM community. Giurgiutiu et al. [31,32,33] investigated an in situ active sensor and embedded self-sensing piezoelectric sensor for online structural health monitoring. Zhu and Qing et al. [34,35] monitored the disbond on patch-bonded repair and honeycomb composite structures. Na et al. covered a newly developed low-cost method for adhesive debonding detection with the EMI technique and monitored metal-based pipeline facilities [36,37]. Some investigations focused on the mechanical performance of single-lap joints, but only using the quasi-static methodology. These studies included the external low-velocity impact or fatigue performance [38,39]. There were also some other investigations focusing on the monitoring of civil engineering structures. Gu et al. [40] studied the multi-functional smart aggregate-based structural health monitoring of circular reinforced concrete (RC) columns subjected to seismic excitations. Karayannis et al. [41] investigated the experimental damage evolution of reinforced concrete steel bars using piezoelectric sensors. Hu et al. [42] reported damage detection with electromechanical impedance method on a concrete slab structure. Voutetaki et al. [43] studied a new experimental method for damage identification of RC beams with piezoelectric transducers. Chalioris et al. [44] and Perera [45] et al. separately investigated the application of wireless monitoring systems to structures in the field of SHM.

However, only few investigations on single-lap joints with the EMI technique were conducted [46,47], especially on the issue of quasi-static behavior online monitoring. 

In order to solve the abovementioned issue to a certain extent, a novel active monitoring scheme based on the EMI technique is proposed in this paper to evaluate the integrity and performance of single-lap joints of fiber-reinforced composites, using square a PZT and the root-mean-square deviation (RMSD) index. A group of effective structural mechanical impedance (ESMI) signatures are extracted from the measured raw signatures in the first place, with the ESMI extraction method referring to the work by Bhalla et al. [29,30]. Then, the reduced-ESMI (R-ESMI) signal processing method is subsequently developed on the basis of the ESMI method. For the purpose of verifying the effectiveness of the proposed monitoring scheme, the following three objectives are proposed: (1) obtaining the ESMI and R-ESMI signatures from the measured raw signatures, (2) monitoring the quasi-static response behavior of single-lap joints of fiber-reinforced composites under the action of tensile displacement, and (3) intuitively characterizing the property variation of single-lap joints with an RMSD index based on the raw signatures, ESMI signatures, and R-ESMI signatures.

## 2. Theoretical Principles

The abovementioned coupled electromechanical admittance expression for a square PZT derived by Bhalla et al. [29,30] is shown in Equation (1).

(1) $$\bar{Y}=G+j\cdot B=j\omega \frac{4l^{2}}{h}\left[\bar{\varepsilon_{33}^{T}}-\frac{2d_{31}^{2}\bar{Y_{11}^{E}}}{\left(1-\nu \right)}+\frac{2d_{31}^{2}\bar{Y_{11}^{E}}}{\left(1-\nu \right)}\left(\frac{Z_{a,eff}}{Z_{a,eff}+Z_{s,eff}}\right)\bar{T}\right],$$

where the meaning of variables is listed in the nomenclature section.

The expression of $\bar{T}$ can be defined in two different cases [30], as shown in Equation (2).

(2) $$\bar{T}=\left\{ \begin{array}{l} \frac{\tan\left(A\kappa l\right)}{A\kappa l}\;\mathrm{for}\;\sin\mathrm{gle}-\mathrm{peak}\;\mathrm{behavior}\;\\ \frac{1}{2}\left[\frac{\tan\left(A\kappa l\right)}{A\kappa l}+\frac{\tan\left(B\kappa l\right)}{B\kappa l}\right]\;\mathrm{for}\;\mathrm{twin}-\mathrm{peak}\;\mathrm{behavior} \end{array}\right..$$

According to the Bhalla’s investigation [30], the expression of pure mechanical impedance of PZT is shown in Equation (3).

(3) $$Z_{a,eff}=\frac{2h\bar{Y_{11}^{E}}}{j\omega \left(1-\nu \right)\bar{T}}.$$

In order to make the expression more intuitive, a simplified form of the admittance coupling equation is deduced on the basis of Equation (1), which endows the equation with a more lucid physical meaning. The modified admittance coupling equation can be rewritten as another expression that is made up of two components.

(4) $$\bar{Y}=\underset{Component\;I}{\underset{︸}{j\omega \bar{C}\cdot \left(1-{\bar{k}}_{p}^{2}\right)}}+\underset{Component\;II}{\underset{︸}{j\omega \bar{C}\cdot {\bar{k}}_{p}^{2}\left(\frac{Z_{a,eff}}{Z_{a,eff}+Z_{s,eff}}\right)\bar{T}}},$$

where $\bar{C}=\varepsilon_{33}^{T}{\left(2l\right)}^{2}/h$, and $\bar{k_{p}^{2}}=2d_{31}^{2}\bar{Y_{11}^{E}}/\varepsilon_{33}^{T}\left(1-\nu \right)$. The meaning of each symbol is listed in nomenclature section in detail.

From Equation (4), it can be clearly seen that the first part of coupled electromechanical admittance (component I) is only dependent upon the parameters of PZT, while the second part of admittance (component II) is partly related to the structural mechanical impedance. Thus, component II can be used for evaluating the damage occurrence and propagation in the structures. Hence, component I and component II can be separately defined as a “non-damage-sensitive item” (${\bar{Y}}_{nons}$) and a “damage-sensitive item” (${\bar{Y}}_{sens}$), and Equation (4) can be concisely written as

(5) $$\bar{Y}={\bar{Y}}_{nons}+{\bar{Y}}_{sens}.$$

The expression of ${\bar{Y}}_{nons}$ is given in Equation (6).

(6) $${\bar{Y}}_{nons}=G_{nons}+jB_{nons}=j\omega \bar{C}\cdot \left(1-{\bar{k}}_{p}^{2}\right),$$

where

(7) $$\left\{ \begin{array}{l} G_{nons}=\omega \frac{{\left(2l\right)}^{2}}{h}\left[\delta \varepsilon_{33}^{T}+\frac{2d_{31}^{2}Y_{11}^{E}\eta }{\left(1-\nu \right)}\right]\\ B_{nons}=\omega \frac{{\left(2l\right)}^{2}}{h}\left[\varepsilon_{33}^{T}-\frac{2d_{31}^{2}Y_{11}^{E}}{\left(1-\nu \right)}\right] \end{array}\right..$$

According to Equation (5), ${\bar{Y}}_{sens}$ can be given by

(8) $${\bar{Y}}_{sens}=\bar{Y}-{\bar{Y}}_{nons}.$$

Hence, the expressions of the real part and imaginary part can be easily obtained, as shown in Equation (9).

(9) $$\left\{ \begin{array}{l} G_{sens}=G-G_{nons}\\ B_{sens}=B-B_{nons} \end{array}\right..$$

According to Equations (4) and (5), the expression of ${\bar{Y}}_{sens}$ is given by

(10) $${\bar{Y}}_{sens}=j\omega \bar{C}\cdot {\bar{k}}_{p}^{2}\left(\frac{Z_{a,eff}}{Z_{a,eff}+Z_{s,eff}}\right)\bar{T}.$$

Assuming $\bar{T}=\alpha +j\beta$ and then substituting the expressions of $\bar{C}$ and ${\bar{k}}_{p}^{2}$ into Equation (10), Equation (11) can be obtained. 

(11) $${\bar{Y}}_{sens}=G_{sens}+jB_{sens}=j\omega \cdot \frac{{\left(2l\right)}^{2}}{h}\cdot \frac{2d_{31}^{2}Y_{11}^{E}\left(1+j\eta \right)}{\left(1-\nu \right)}\cdot \left(\frac{Z_{a,eff}}{Z_{a,eff}+Z_{s,eff}}\right)\cdot \left(\alpha +j\beta \right).$$

By rearranging the items in Equation (11), a new formula can be written as

(12) $$\gamma_{1}+j\cdot \tau_{1}=\left(\gamma_{2}+j\cdot \tau_{2}\right)\cdot \frac{Z_{a,eff}}{Z_{a,eff}+Z_{s,eff}},$$

where

(13) $$\left\{ \begin{array}{l} \lambda =\frac{8\omega l^{2}d_{31}^{2}Y_{11}^{E}}{h\left(1-\nu \right)}\\ \gamma_{1}=\frac{1}{\lambda }B_{sens}=\frac{1}{\lambda }\left(B-B_{nons}\right)\\ \tau_{1}=-\frac{1}{\lambda }G_{sens}=-\frac{1}{\lambda }\left(G-G_{nons}\right)\\ \gamma_{2}=\alpha -\eta \beta \\ \tau_{2}=\alpha \eta +\beta \end{array}\right..$$

Furthermore, defining $Z_{s,eff}=x_{str}+j\cdot y_{str}$ and $Z_{a,eff}=x_{a}+jy_{a}$, solving Equation (12) yields

(14) $$\left\{ \begin{array}{l} x_{str}=\left(\frac{\gamma_{1}\gamma_{2}+\tau_{1}\tau_{2}}{\gamma_{1}^{2}+\tau_{1}^{2}}-1\right)x_{a}-\left(\frac{\gamma_{1}\tau_{2}-\gamma_{2}\tau_{1}}{\gamma_{1}^{2}+\tau_{1}^{2}}\right)y_{a}\\ y_{str}=\left(\frac{\gamma_{1}\tau_{2}-\gamma_{2}\tau_{1}}{\gamma_{1}^{2}+\tau_{1}^{2}}\right)x_{a}+\left(\frac{\gamma_{1}\gamma_{2}+\tau_{1}\tau_{2}}{\gamma_{1}^{2}+\tau_{1}^{2}}-1\right)y_{a} \end{array}\right..$$

Equation (14) is simpler in the form of expression than that derived by Bhalla [30]. With the use of Equations (13) and (14), the required signatures can be extracted from the raw signatures. It must be mentioned that, according to the derivation of Equations (12)–(14), the self-impedance of the square PZT sensor is subtracted to acquire the $x_{str}$ and $y_{str}$ signatures, which results in too many negative ESMI signatures. To solve this problem and ensure more signatures are positive in the coordinate system, as well as making the scale of vertical coordinates identical, another two variables $x_{s}$ and $y_{s}$ are defined, and they are opposite in number to $x_{str}$ and $y_{str}$, as shown in Equation (15).

(15) $$\left\{ \begin{array}{l} x_{s}≜-x_{str}/10^{4}\\ y_{s}≜-y_{str}/10^{4} \end{array}\right..$$

In order to monitor the quasi-static response behavior of a single-lap joint, a very effective and commonly used statistical index RMSD is introduced. 

The novel statistical index of RMSD used in this paper is given by Equation (16).

(16) $$RMSD=\sqrt{\frac{\sum_{t=1}^{N}{\left(D_{t}-B_{t}\right)}^{2}}{\sum_{t=1}^{N}B_{t}^{2}}},$$

where the symbol N denotes the number of data points, *t* is the counter, $D_{t}$ represents the $t^{th}$ data point acquired in damage state, and $B_{t}$ is the $t^{th}$ data point acquired in the healthy state. 

With the use of the above-deduced equations, the EMSI signatures can be obtained. However, based on the EMSI, a further modified method, R-EMSI, can be obtained. R-EMSI includes two similar ways of signal processing. Firstly, R-EMSI considers the signatures at peaks, and then these signatures are adopted for calculating the values of RMSD. Secondly, R-EMSI only considers the signatures at the main peak (the first peak), and then the signatures at the main peak are used for calculating the same damage indicator. The methodology of EMSI is able to reduce the scale of signature processing while the R-EMSI reduces the data scale further. Both methods of EMSI and R-EMSI are investigated in detail in the later sections.

## 3. Experimental Set-Up

In order to study the effectiveness of the methodology presented above, a verifying experiment was conducted.

**Figure 1:**
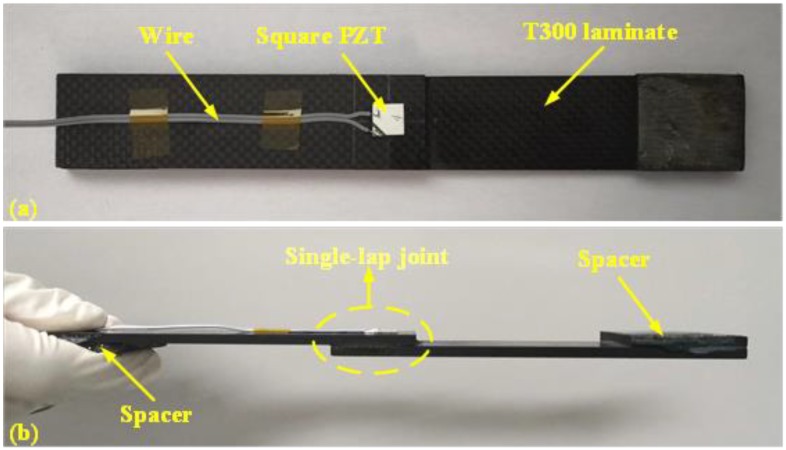
Standard specimen for tensile test: (**a**) top view; (**b**) side view.

Referring to ASTM D5868, a specimen of a composite single-lap joint was fabricated using a T300 woven prepreg with vacuum bag molding (VBM) [48]. Then, a square PZT sensor was mounted on the surface of the joint area with high-strength epoxy resin and wired to acquire the raw signatures. Although better monitoring results can be obtained with the PZT sensor network in terms of many investigations, it should be mentioned that the size of the specimen is very small; thus, only a PZT sensor was mounted on the most concerned the joint area. The key parameters of the T300 woven prepreg and square PZT sensor are separately enumerated in Table 1 and Table 2. The prepared specimen is shown in Figure 1, and it should be mentioned that the initial gauge length of the specimen was 150 mm.

**Figure 2:**
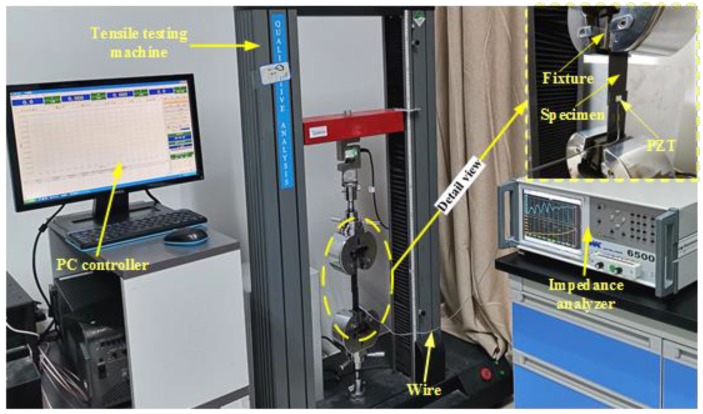
Testing platform for single-lap joint quasi-static behavior monitoring.

The displacement load was applied to the standard specimen using the tensile testing machine. In the meantime, an impedance analyzer (WK-6500B, Wayne Kerr Electronics, London, UK) was connected to the PZT to acquire the raw conductance and susceptance signatures in real time. The integrated testing system for single-lap joint quasi-static behavior monitoring is shown in Figure 2.

During the process of the experiment, 19 response states (from N-1 to N-19) were defined under the action of uniaxial tensile displacement, and their pertinent raw signatures were obtained through the impedance analyzer. The signatures acquired in the initial state of N-1 were selected as the baseline. N-19 represented the state at the moment of single-lap joint separation (fracture). The displacement increasing rate was 0.1 mm/min at each testing state, as listed in Table 3. The frequency scope selected for the experiment was from 500 kHz to 2.5 MHz with a step of 1.25 kHz. The reason for selecting such a high frequency scope was that the joint region of the specimen was small and it had better sensitivity with higher frequency.

**Figure 3:**
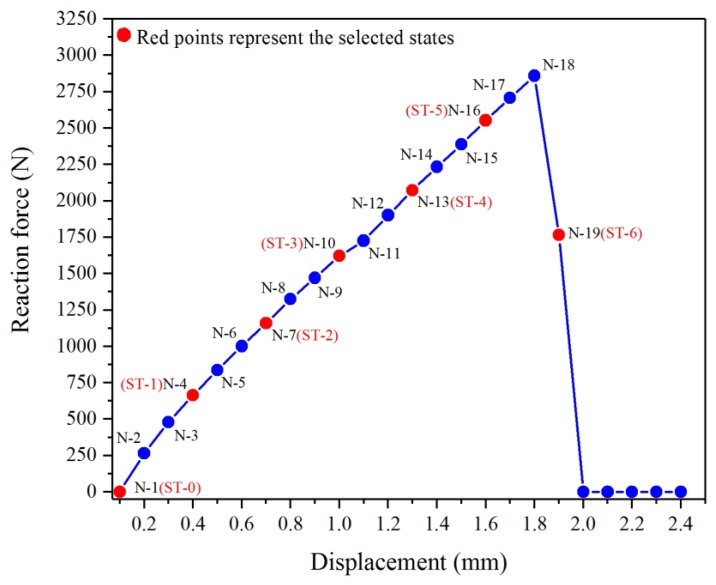
“Displacement vs. reaction force” curve in quasi-static tensile experiment.

Based on the correlation between applied displacement and measured reaction force, the curve graph of “displacement vs. reaction force” could be plotted after amending the rigid body displacement, as shown in Figure 3. Although the raw signatures of the 19 testing states were obtained in the process of the experiment, in order to investigate the correlation between the quasi-static response of the single-lap joint and tensile displacement more lucidly, we selected seven typical states with a uniform interval of every two points (states) and then renamed these selected states from ST-0 to ST-6. The selected states are marked with red color on the curve in Figure 3. These selected and renamed states for subsequent comparison and discussion are enumerated in Table 4.

Eventually, the raw signatures of the seven selected representative states were processed with the methodology presented in Section 2. The obtained ESMI signatures were then used for evaluating the quasi-static response behavior in the process of the tensile experiment. The experimental results related to the single-lap joint are discussed in detail in the next section.

## 4. Results and Discussion

### 4.1. Quasi-Static Behavior of Single-Lap Joint Analysis

**Figure 4:**
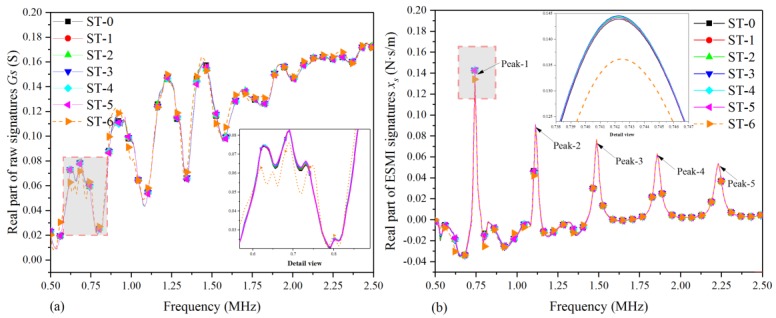
Real part plot of raw and effective structural mechanical impedance (ESMI) signatures: (**a**) G_s_; (**b**) x_s_.

**Figure 5:**
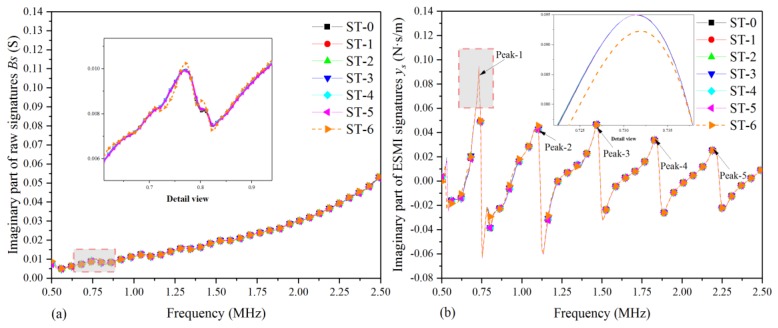
Imaginary part plot of raw and ESMI signatures: (**a**) B_s_; (**b**) y_s_.

In light of Section 2 and Section 3 of the paper, the raw signatures were processed with the ESMI methodology. Then, the curve graphs of Figure 4 and Figure 5 were plotted to compare the sensitivity to quasi-static characteristics of the single-lap joint before and after signature extraction. In addition, in order to investigate the regularity of the quasi-static behavior of the single-lap joint with respect to the tensile load in a clearer way, only seven typical states were selected, as listed in Table 4.

Figure 4 shows the graph plotted based on the real part of the raw signatures and ESMI signatures of the seven selected typical states. From Figure 4a, it can be clearly seen that there was an increasing tendency of curves in all selected states, and no obvious peaks could be selected as the feature peak for characterizing the quasi-static response behavior under the action of external load. On the contrary, using the above-presented ESMI methodology for signal processing, a group of more effective signatures could be obtained, and five characteristic peaks appeared, which could then be used for investigating the serving condition of the single-lap joint, as shown in Figure 4b.

Moreover, from Figure 4b, it can be also found that the amplitude of the curve in each state changed gradually with the increase in tensile displacement, and there were slight shifts in peak frequency in each state. With the increase in tensile displacement, there were also gradual changes in signature amplitude, but the amplitude dropped sharply at the moment of joint separation, which was intuitively observed.

Figure 5 shows the graph plotted based on the imaginary part of raw and ESMI signatures for the abovementioned seven selected typical states. From Figure 5a, it can be obviously seen that there was a rising tendency of curves in these selected representative states, and no obvious peaks could be used to act as characteristic peaks for investigating the quasi-static response behavior with respect to the tensile displacement. However, using the abovementioned ESMI methodology for signature extraction, a series of more sensitive signatures could be acquired, and several new characteristic peaks appeared, which could then be used for supplementing the information on the service condition monitoring of the single-lap joint, as shown in Figure 5b.

Moreover, in Figure 5b, it can also be observed that the amplitude of the curve in each state changed gradually with the increase in tensile displacement, and there were slight shifts in peak frequency in each state. At the beginning of the tensile experiment, the amplitude gradually increased with the displacement, and the amplitude dropped sharply when joint separation occurred, albeit not as obvious as that in the real part case. 

### 4.2. Characterization of Single-Lap Joint Condition with RMSD Index

Despite the curves in Figure 4 and Figure 5 showing the correlation between tensile displacement and measured signatures, no intuitive enough regularity could be found. Hence, to solve this problem, the index of RMSD was adopted. 

**Figure 6:**
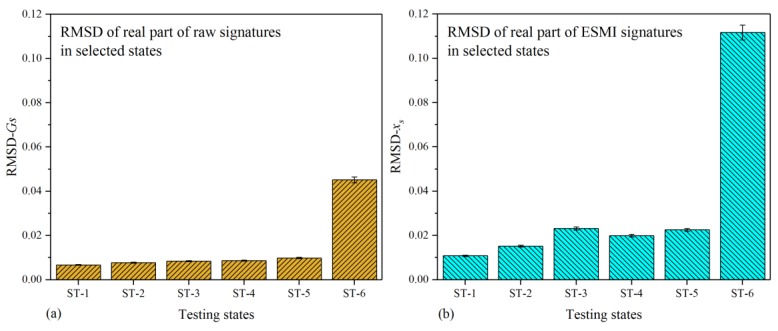
Histogram of real part of raw and ESMI signatures: (**a**) G_s_; (**b**) x_s_.

**Figure 7:**
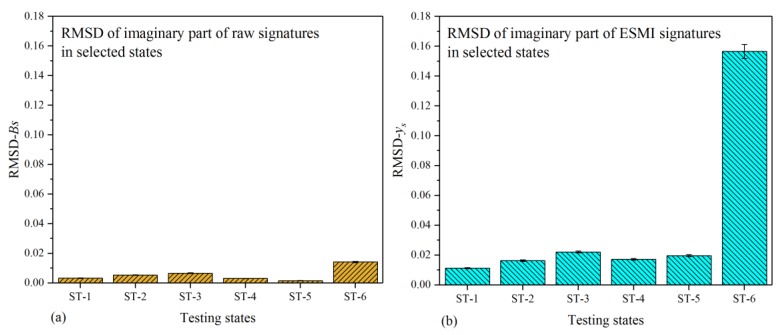
Histogram based on imaginary part of raw and ESMI signatures: (**a**) B_s_; (**b**) y_s_.

As mentioned above, the curves in Figure 4b and Figure 5b show very obvious peaks (Peak-1 to Peak-5); thus, a novel signature processing method was developed based on the extracted ESMI signatures, which involved calculating the RMSD index with the peak ESMI signatures of the selected typical states. The RMSD values were then calculated on the basis of these obtained peak values, as well as those based on raw signatures. These RMSD histograms are shown in Figure 6 and Figure 7. It should be pointed out that the magnitudes of histograms in the vertical coordinate were identical so as to scale the discrepancy between RMSD bars. 

Figure 6 shows the histograms plotted based on the real part of the raw and ESMI signatures of the single-lap joint. It can be clearly seen that the largest magnitude of RMSD occurred in the state of ST-6 among the selected testing states, both in Figure 6a,b. Due to the real part having excellent sensitivity to structural deterioration, the pertinent raw signatures and extracted ESMI signatures showed good effectiveness when used for studying the variation characteristics of the single-lap joint. Therefore, the real part of both the ESMI signatures and raw signatures could be used for evaluating the health condition of the joint structure. Nevertheless, it should be pointed out that the difference between ST-6 and other states (ST-1 to ST-5) in Figure 6b was much larger than that in Figure 6a. Hence, the histogram of *x_s_* was capable of characterizing the correlation between displacement and the RMSD index in a more effective manner. 

Figure 7 shows the histograms plotted based on the imaginary part of the raw and ESMI signatures. It is easily noted that the largest RMSD bars in magnitude appeared in the state of ST-6, which denoted the moment of joint separation, both in Figure 7a,b. According to the experimental investigation, we considered the imaginary part of the raw signatures to have better sensitivity to reaction force variation. In terms of Figure 3, the reaction force initially increased with the tensile displacement, then decreased when the bearing capability of the structure worsened. This characteristic could also be indicated with the impedance (or admittance) index.

Upon processing using the ESMI methodology, a group of signatures more sensitive to structural variation than to reaction force variation could be extracted, as shown in Figure 7b. According to the states from ST-1 to ST-6, the changing law monotonically increased, and the difference between two bars was more distinct in Figure 7b. Hence, from the authors’ viewpoint, the histogram of *y_s_* was also competent for effectively indicating the correlation between displacement and the RMSD index.

### 4.3. Data Processing Scale Reduction Method for Single-Lap Joint Monitoring

In the preceding section, for the purpose of comparing and analyzing the experiment results in a more concise and lucid manner, only some typical states were selected; however, as is well known, better investigation results can be obtained if more testing states are taken into consideration. However, more testing states bring in a mass of experimental data to deal with and this process is usually complicated; thus, a novel method called R-ESMI was developed to reduce the data processing scale. 

R-ESMI is an improved methodology of ESMI, aimed at obtaining the peak values from the extracted ESMI signatures before using them to calculate the RMSD index to investigate the quasi-static characteristics of single-lap joints. The R-ESMI method includes two methods to reduce the processing scale of data: (1) calculating RMSD values based on all of the ESMI peak signatures (from Peak-1 to Peak-5), or (2) based on the main peak signatures only (Peak-1). The pertinent histograms were plotted and compared in terms of the above RMSD values of raw signatures, ESMI signatures, and R-ESMI signatures.

**Figure 8:**
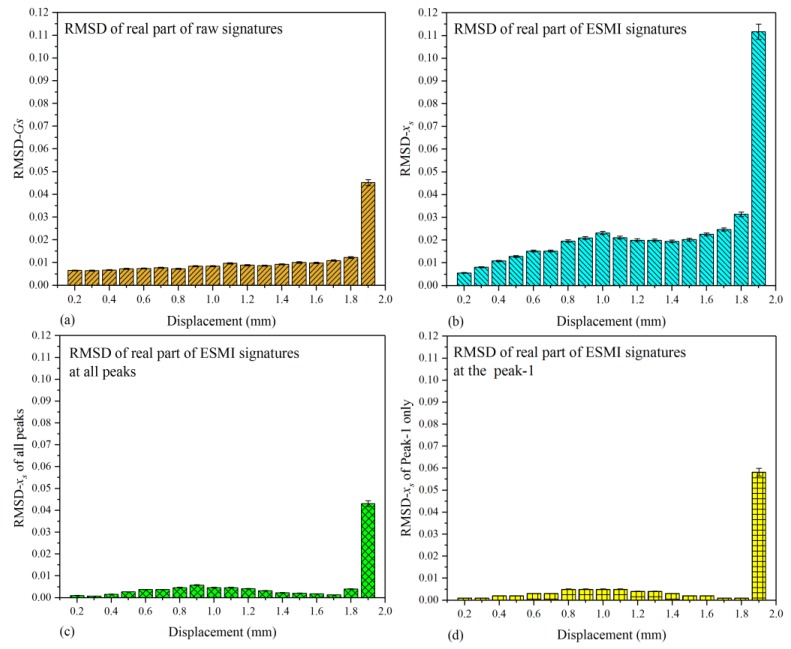
Comparative root-mean-square deviation (RMSD) histograms based on real part of signatures in all testing states: (**a**) raw G_s_ signatures; (**b**) extracted x_s_ signatures; (**c**) extracted x_s_ signatures at all peaks; (**d**) extracted x_s_ signatures at Peak-1 only.

For the purpose of characterizing the quasi-static behavior of the single-lap joint in a more effective way, the RMSD values from N-1 to N-19 (all testing states) were calculated, and their pertinent histograms were plotted. These obtained RMSD values were separately based on the real parts of raw signatures, ESMI signatures, and R-ESMI signatures (ESMI signatures at peaks from Peak-1 to Peak-5 and signatures at Peak-1 only). The corresponding histograms were then comparatively plotted, as shown in Figure 8.

**Figure 9:**
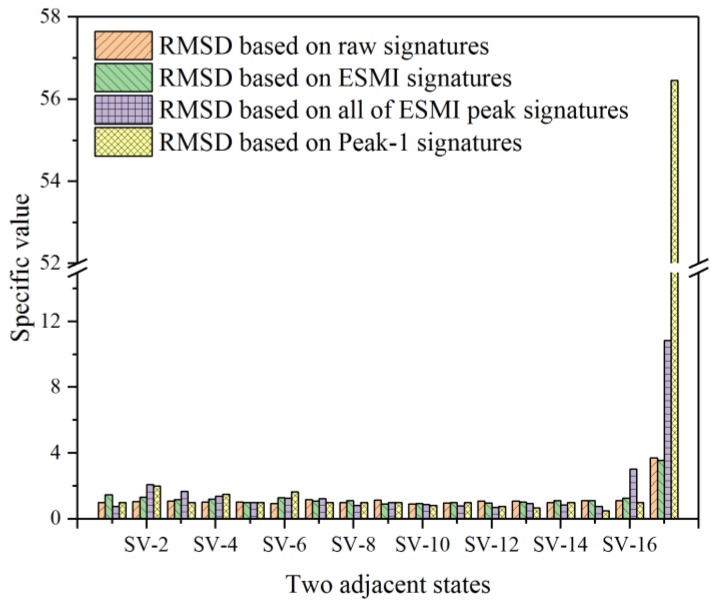
Histogram of specific values of two adjacent RMSD bars based on real part signatures.

From Figure 8a–d, it can be easily noted that there were differences in bar height among testing states in the four figures. In order to quantify this difference in a more intuitive way, specific values (SV) of every two adjacent RMSD bars in different testing states were separately calculated using Equation (17). The histogram of these specific values was then plotted, as shown in Figure 9.

(17) $$SV_{k-1}=\frac{{\left(RMSD\right)}_{k}}{{\left(RMSD\right)}_{k-1}}\;(k=2,\;3,\;4\cdots 19),$$

where *k* is the sequence number of the bar in the RMSD histogram.

Figure 9 shows a very obvious difference among the four types of RMSD bars, as annotated in the figure. It indicates that the calculated RMSD values based on the Peak-1 signatures showed the best effectiveness when used for characterizing the quasi-static behavior of the single-lap joint, followed by the RMSD calculated on the basis of ESMI signatures of all peaks. 

**Figure 10:**
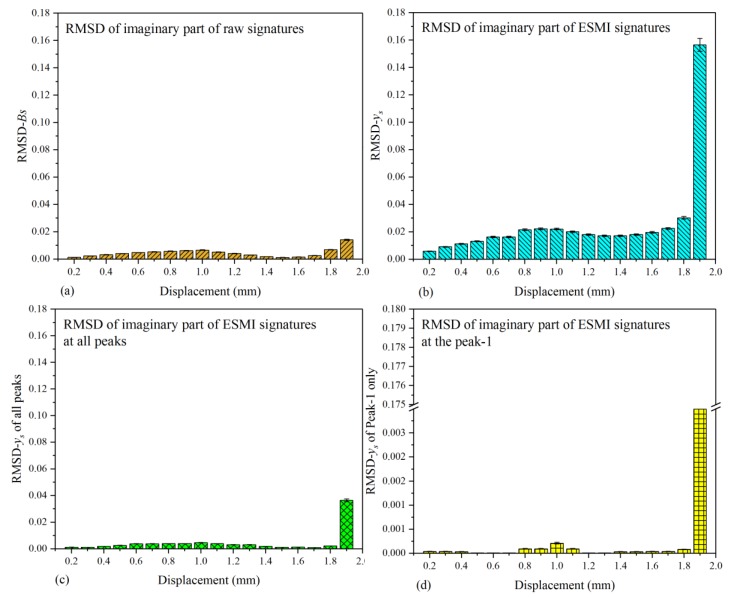
Comparative RMSD histograms based on imaginary part of signatures in all testing states: (**a**) raw G_s_ signatures; (**b**) extracted x_s_ signatures; (**c**) extracted x_s_ signatures at all peaks; (**d**) extracted x_s_ signatures at Peak-1 only.

Apart from characterizing the regular pattern of the single-lap joint with the real parts of signatures, the imaginary ones are also needed to provide additional information to study this issue comprehensively. Hence, the RMSD values based on the imaginary parts of signatures were also calculated, and the pertinent RMSD histogram is illustrated in Figure 10. 

As shown in Figure 10a, it can be deduced that the deviation of N-2 to N-10 from baseline became larger and the impedance (or admittance) increased, which indicates that the single-lap joint was capable of bearing a larger reaction force at these moments. Subsequently, when the joint was stretched to a certain extent, 1.0 mm in the experiment, the load bearing capacity of the joint began to deteriorate. The impedance (or admittance) variation of the joint structure in amplitude decreased and, when the tensile displacement was 1.5 mm, the impedance (or admittance) of the joint structure lowered to the baseline state (N-1). Subsequently, the impedance (or admittance) continued lowering. Then, the deviation of impedance (or admittance) between the current state and baseline state went downward, which led to the RMSD index becoming larger again.

In order to reduce the data processing scale, the effectiveness of the ESMI and R-ESMI methodologies was compared. The RMSD graphs on the basis of ESMI and R-ESMI signatures are shown in Figure 10b–d. In Figure 10b, the variation tendency of RMSD bars was similar to that in Figure 8b, which indicates that the sensitivity of RMSD-*y_s_* to reaction force declined and became more sensitive to the tensile displacement variation, which can be deduced through carefully comparing Figure 10b with Figure 8a,b. However, from the authors’ perspective, there are still too many ESMI signatures to be processed. Hence, R-ESMI was subsequently developed to solve this problem further. 

As described above, the R-ESMI methodology includes two ways to calculate the RMSD index, based on all peak signatures (signatures at the peak from Peak-1 to Peak-5) or the main peak (Peak-1) signatures only. The histograms are shown in Figure 10c,d. 

In Figure 10c, the difference among the bars in height became larger than that in Figure 10a,b. Furthermore, the calculated RMSD values on the basis of the main peak (Peak-1) signatures were also plotted, as shown in Figure 10d. Some bar heights were too small to be displayed compared with the bar representing the state of joint separation. Moreover, by comparing the four subfigures in Figure 10, a conclusion can be drawn that the largest difference among bars was in Figure 10d, which proves that the method of R-ESMI with signatures at Peak-1 only had the best effectiveness in reducing the data processing scale. 

**Figure 11:**
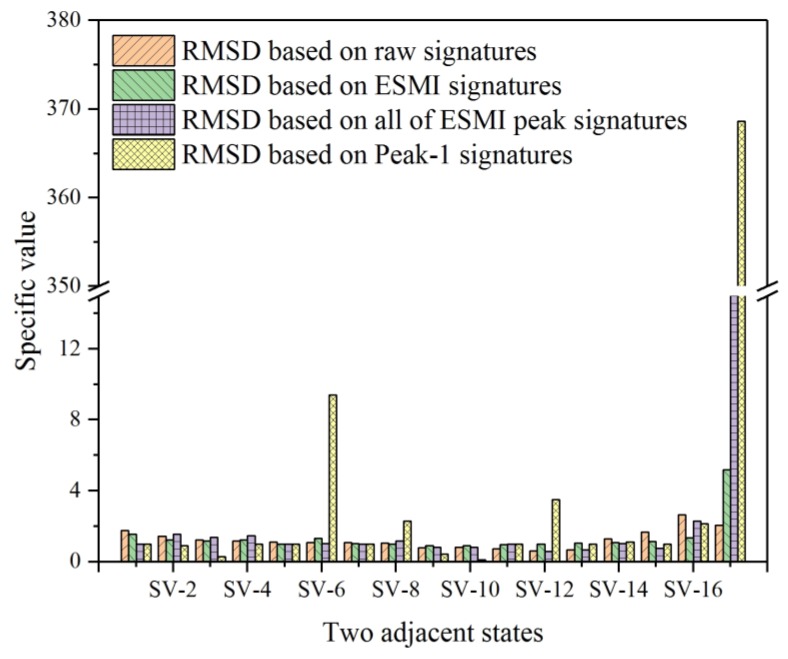
Histogram of specific values of two adjacent RMSD bars based on imaginary part signatures.

For the purposing of evaluating this discrepancy among bars more directly, the specific values between every two RMSD bars in Figure 10 were also calculated using Equation (17). Then, the corresponding histogram was plotted, as shown in Figure 11. In this figure, the difference between the two adjacent RMSD bars could be quantified, supporting the aforementioned conclusion of the R-ESMI (Peak-1 signatures only) having the best monitoring effectiveness with the least signal processing scale. Furthermore, another obtained inference was that the imaginary parts of signatures can be also adopted to evaluate the structural health condition if an appropriate signal processing method is applied.

## 5. Conclusions

In this paper, ESMI and R-ESMI methodologies for single-lap joint quasi-static behavior monitoring were presented, aimed at studying the quasi-static response of this joint structure in a more accurate but simpler way. The two methodologies, ESMI and R-ESMI, were developed from a modified two-dimensional EMI model for square PZT sensors. 

The intuitive characterization of the joint’s health condition was performed via the variation in RMSD index, whose values were calculated on the basis of raw signatures, ESMI signatures, and R-ESMI signatures. Additionally, the specific value (SV) was also calculated to quantify the difference between two adjacent RMSD bars in magnitude, which also contributed to demonstrating the effectiveness of the methodologies. Using the methodology presented in this article, obvious peaks were successfully extracted from the raw signatures with the use of ESMI methodology, which proves its potential application in structural health monitoring of fiber-reinforced composite structures. Then, the quasi-static response behavior of the single-lap joint could be characterized intuitively and effectively via combining the ESMI and R-ESMI signatures with the RMSD index. By adopting the methodology developed in this paper, the conventionally ignored susceptance signatures can be also used for evaluating the health condition of a single-lap joint of fiber-reinforced composites if processed with ESMI and R-ESMI methodologies. 

The characterization of quasi-static behavior using the electromechanical impedance method is difficult, and more work is needed to study this issue further in our future work. The strain rate needs to be increased to simulate the obvious variation of external loads in a short time. The initial damage inside the composite-lap joint should be analyzed. The smallest damages that can be monitored in quasi-static or dynamic processes should be studied. The impacts of is the environment also need investigating.

## Figures and Tables

**Table 1:** Key parameters of T300 woven prepreg.

Elastic constants	Physical parameters	Values
*E*_1_ (GPa)	Young’s modulus *X*-direction	61.34
*E*_2_ (GPa)	Young’s modulus *Y*-direction	61.34
*E*_3_ (GPa)	Young’s modulus *Z*-direction	6.9
*ν*_12_	Poisson’s ratio *XY*	0.04
*ν*_23_	Poisson’s ratio *YZ*	0.3
*ν*_13_	Poisson’s ratio *XZ*	0.3
*G*_12_ (GPa)	Shear modulus *XY*	19.5
*G*_23_ (GPa)	Shear modulus *YZ*	2.7
*G*_13_ (GPa)	Shear modulus *XZ*	2.7

**Table 2:** Key parameters of square piezoelectric transducer (PZT) sensor.

Parameters	Physical parameters	Values
$Y_{11}^{E}\left(\times 10^{10}N/m^{2}\right)$	Young’s modulus at constant electric field	9.3
η	Mechanical loss factor	0.01
$\varepsilon_{33}^{T}/\varepsilon_{0}$	Relative permittivity at constant stress	1920
δ	Dielectric loss factor	0.025
$d_{31}\left(C/N\right)$	Piezoelectric strain coefficient	−200
ν	Poisson’s ratio	0.32
$\rho \left(kg/m^{2}\right)$	Density	7750
h(mm)	Thickness	0.33
2l(mm)	Length	10

**Table 3:** Testing states in tensile experiment.

Testing States	Displacement (mm)	Testing States	Displacement (mm)
N-1 (baseline)	0.1	N-11	1.1
N-2	0.2	N-12	1.2
N-3	0.3	N-13	1.3
N-4	0.4	N-14	1.4
N-5	0.5	N-15	1.5
N-6	0.6	N-16	1.6
N-7	0.7	N-17	1.7
N-8	0.8	N-18	1.8
N-9	0.9	N-19 (joint separation)	1.9
N-10	1.0		

**Table 4:** Selected and renamed typical states.

Selected Testing States	Displacement (mm)	Notes
ST-0	0.1	N-1 in original state
ST-1	0.4	N-4 in original state
ST-2	0.7	N-7 in original state
ST-3	1.0	N-10 in original state
ST-4	1.3	N-13 in original state
ST-5	1.6	N-16 in original state
ST-6 (joint separation)	1.9	N-19 in original state

## Nomenclature

j	Complex symbol
ω	Angular frequency (rad·s^−1^)
C	Capacitance symbol (F)
$\varepsilon_{33}^{T}$	Dielectric constant (F·m^−1^)
2l	Length of the square PZT (mm)
h	Thickness of circular PZT (mm)
$k_{p}$	Coupling coefficient
$d_{31}$	Piezoelectric strain constant (PC·N^−1^)
$Y_{11}^{E}$	Young’s modulus (N·m^−2^)
ν	Poisson ratio
κ	Wavenumber (m^−1^)
$c_{p}$	Phase velocity (m·s^−1^)
$Y_{nons}$	Damage-non-sensitive component in admittance model (S)
$Y_{sens}$	Damage-sensitive component in admittance model (S)
Y	Coupling mechanical admittance (S)
ρ	Density (kg·m^−3^)
η	Mechanical loss factor
δ	Dielectric loss factor
$Z_{a,eff}$	Effective driving point impedance of PZT under short-circuited condition (N·s/m)
$Z_{s,eff}$	Effective driving point impedance of host structure (N·s/m)
Z	Coupling mechanical impedance (Ω)
$x_{str}$	Real part of ESMI signatures
$y_{str}$	Imaginary part of ESMI signatures
*SV*	Specific value of two RMSD values in adjacent testing states
